# Remembering what did not happen: the role of hypnosis in memory recall and false memories formation

**DOI:** 10.3389/fpsyg.2025.1433762

**Published:** 2025-02-04

**Authors:** Donato Giuseppe Leo, Davide Bruno, Riccardo Proietti

**Affiliations:** ^1^Department of Cardiovascular and Metabolic Medicine, Institute of Life Course and Medical Sciences, Faculty of Health and Life Sciences, University of Liverpool, Liverpool, United Kingdom; ^2^Liverpool Centre for Cardiovascular Science, University of Liverpool and Liverpool Heart and Chest Hospital, Liverpool, United Kingdom; ^3^School of Psychology, Liverpool John Moores University, Liverpool, United Kingdom

**Keywords:** false memories, hypnosis, memory, pseudo-memories, memory recall

## Abstract

Memory recall is subject to errors that can lead to the formation of false memories. Several factors affect memory processes, such as attention deficits or emotional distress. Additionally, cardiovascular diseases may lead to cognitive decline and memory loss, also increasing the occurrence of false events recall. Hypnosis has proved to affect the autonomic nervous system, positively impacting the cardiovascular response. Hypnosis has also been suggested as a tool to enhance memory and autobiographical events recall in both healthy and unhealthy individuals; however, this approach has led to several controversies. Particularly, the employment of hypnosis in autobiographical recall (hypnotic regression) has been accused of favoring the creation of false memories, leading to therapeutic fallacy. In this paper, we review the current literature on the mechanisms behind the creation of false memories and the role played by hypnosis in memory enhancement and false memory recall. The evidence here collected suggests that cardiovascular diseases affect brain health contributing to cognitive decline and memory impairments, also increasing the occurrence of false memories. Hypnosis induces an increase in parasympathetic activity and a decrease in sympathetic activity, suggesting a potential role in preventing some cardiovascular diseases, such as hypertension, which in turn may improve brain health. Additionally, hypnosis has been shown to have some effectiveness in enhancing memory functions, although contradictory findings reported by several studies make it difficult to draw proper conclusions. Hypnotic regression and guided imagery should be used with caution as they may unintentionally lead to false memory recall. Nevertheless, further studies are required to better understand the effects of hypnosis on the brain and the heart and how it can be used to enhance memory, especially in people with cognitive decline.

## Introduction

Memory is the faculty of the mind that encodes, stores and retrieves information, and it is fundamental in the development of personal identity (Baddeley, 2013). While some degree of forgetting in general is part of normal memory function (Jasnow et al., 2012; Murayama et al., 2014; Williams et al., 2013), significant memory deficits may be related to age-related cognitive decline (e.g., dementia; Jahn, 2013), emotional or physical trauma (Van Der Kolk, 1998), or interference in memory processing due to poor mental health (such as depression, attention deficit, or emotional distress; Martinussen et al., 2005; Tyng et al., 2017). However, in contrast to forgetting an event, it is also possible to remember it incorrectly, typically leading to false memories (Martinussen et al., 2005).

Several theories have been proposed to explain false memory formation: of these, some attribute it to the way in which information is stored (fuzzy-trace theory; Reyna, 2013), and to the malleability of memory (construction hypothesis; Loftus, 1975). Additional factors may influence the formation of false memories, such as individual differences (e.g., having greater creativity or a tendency to dissociation; Dehon et al., 2008), social pressure (that increases the tendency to accept false events as true; Reysen, 2007), a history of trauma (that makes people more vulnerable to false memories; Zoellner et al., 2000), and sleep deprivation (that increases the chance of encoding false memories; Diekelmann et al., 2008). Moreover, it is also important to consider the influence that physiological factors have on memory, and how these affect memory recall (Birdsill et al., 2013). The importance of the cardiovascular system in satisfying the oxygen demand of the brain, thus influencing its general health, has been largely discussed in literature (brain-heart interaction; Chen et al., 2017), with cardiovascular diseases negatively affecting cognition and memory (e.g., hypertension, heart failure; Cannon et al., 2017; Feng et al., 2020; Habota et al., 2015; Kalaria et al., 2016; Ungvari et al., 2021).

Hypnosis is an altered state of consciousness characterized by focused attention, reduced peripheral awareness, and an increased tendency to respond to suggestions (Elkins et al., 2015). Hypnosis has been shown to affect the autonomic nervous system (De Benedittis, 2024) and to impact cardiovascular response (Emdin et al., 1996; Yüksel et al., 2013), which in turn may lead to a healthier brain (Kekecs et al., 2016; VandeVusse et al., 2010; Walker et al., 2017). Additionally, hypnosis has been suggested as a tool to enhance memory (hypermnesia) and facilitate memory recall (Mulligan, 2006). For example, hypnotic regression aims to recall the repressed memory of a traumatic experience that occurred in an earlier stage of life (Hunter and Eimer, 2012). However, this practice has been largely criticized, arguing that hypnosis may induce false memories (Bryant and Barnier, 1999) rather than recover forgotten ones, with serious implications in therapy and legal cases (such as the recall of past abuses that never occurred and that the patient now believes as true events; Hyman Jr and Loftus, 2001).

The aims of this narrative review are to: (i) give an overview of the mechanisms behind the formation of false memories, also highlighting the role played by the cardiovascular system, (ii) discuss how hypnosis impacts memory recall and false memories, and (iii) discuss the role of hypnosis in cardiovascular and cognitive functions and its implications for memory enhancement.

## When memory fails: the construction of false memories

Broadly speaking, memory involves short-term processing of both visual and auditory information (working memory), and long-term memory (where information is stored for a long-term period; Baddeley, 2013). Long-term memory processes mainly consist of storage and recollection of information and previous experiences that can be easily verbalized (i.e., declarative memory; e.g., what I ate for breakfast), and of storage and retrieval of non-verbally articulated procedural information (i.e., non-declarative memory; e.g., how to ride a bicycle; Squire and Dede, 2015), generally in reference to the use of objects or to body movements. Declarative memory can be further subdivided into semantic memory (i.e., memory of facts and general knowledge) and episodic memory (i.e., memory of personal events; Greenberg and Verfaellie, 2010). The main cortical areas thought to play a role in memory processes are the prefrontal cortex, considered essential for working memory, complex thought and associative processes (Stuss and Benson, 2019), and the medial-temporal lobe (Davachi and Preston, 2014), including the parahippocampal gyrus (Luck et al., 2010). Subcortically, the hippocampus, also part of the medial-temporal-lobe, is critical for the acquisition and retention of declarative memories (Opitz, 2014), whereas the cerebellum and the basal ganglia are involved in procedural memory (Lee, 2014).

We have known since the work of Frederik Bartlett (Wagoner, 2017) in 1932 that memory does not provide an exact record of experience, but it is rather an adaptive constructive process, which produces errors, distortions, and illusions in order to preserve the functioning of memory itself (Brady et al., 2018; Schacter, 2013; Schacter et al., 2011). Memory is influenced by several factors, including prior knowledge, mood states and the environment in which learning takes place, which may affect the way in which a memory is acquired, stored and eventually reconstructed (El Sharkawy et al., 2008; Long et al., 2008). Attentional narrowing due to extreme stress and strong emotions affects the memory encoding process (Shields et al., 2017), increasing the occurrence of false memories (Kaplan et al., 2016; Van Damme et al., 2017). Episodic memory is more inclined to distortions than semantic memory (Straube, 2012).

Errors in the memory processes (which can lead to forgetting, incorrect recall, or source misattribution; Foley et al., 2015) can occur at the encoding, storage/consolidation, or retrieval stage (Straube, 2012). At the encoding stage, errors leading to false memories can be induced by visual imagery due to overlapping in the encoding of the imagined and perceived events (Gonsalves and Paller, 2000; Gonsalves et al., 2004). At the consolidation stage, errors leading to false memories are due to the interference of previous memories (retroactive interferences) and sleep deprivation (Diekelmann et al., 2008; Zaragoza et al., 2011). At the retrieval stage, errors leading to false memories are due to misinformation provided by retrieval clues (Brainerd and Reyna, 1998). Among false memories, confabulations occur as a compensatory mechanism to fill in gaps in one's memory (Fotopoulou, 2008). This can happen spontaneously (where the false memory is evoked without an external trigger) or be provoked (when the person is prompted to remember a specific event, e.g., a birthday; Kopelman, 1987). Confabulations can be related to several diseases (such as Korsakoff Syndrome, Alzheimer's disease, traumatic brain injury, etc.), but in some cases they can also occur in healthy individuals (Burgess, 1996). The mechanism underlying confabulations has been correlated with brain lesions (mainly located in the prefrontal cortex, frontal lobe and hypothalamus) and with dementia and psychiatric disorders (e.g., schizophrenia; Brown et al., 2017).

Anxiety and depression further increase the occurrence of errors during memory retrieval (Hertel and Brozovich, 2010). The experience of traumatic events may also lead to the formation of false memories, as people tend to remember more trauma that they really experienced (memory amplification effect; Oulton et al., 2016; Strange and Takarangi, 2015). The occurrence of false memories increases with aging, due to the decline in several regions within the medial temporal lobes and the prefrontal cortex (Dennis et al., 2008; Devitt and Schacter, 2016; Fandakova et al., 2018).

A meta-analysis (Kurkela and Dennis, 2016) of neuroimaging studies has shown that the medial superior frontal gyrus and left inferior parietal cortex may play a role in supporting false memory retrieval. Additionally, the weighting of previous knowledge during new memories acquisition by the medial prefrontal cortex may cause interferences during memory retrieval (memory distortion; Berkers et al., 2017). A recent study (Spets et al., 2021) has also shown differences in brain activity between men and women during false memory formation.

The deliberate induction of false memories in someone else's mind (memory implantation) has been deemed possible (Loftus et al., 2014; Loftus and Pickrell, 1995). Techniques to induce memory implantation have been used in cognitive psychology research to demonstrate how unreliable memory can be and to better understand the formation of false memories (Loftus and Pickrell, 1995). Key aspects in false memory formation are: (i) being exposed to misleading information or leading questions (construction hypothesis; Loftus, 1975), (ii) social influence (Reysen, 2007) and personal expectancy (Hirt et al., 1999), and (iii) imagination inflation (where imagining an event that never happened increases the confidence in the veracity of the event; Garry et al., 1996). Psychoactive drugs and sleep deprivation (Kloft et al., 2023), psychotherapeutic practices aiming at memory recall (Loftus, 1996), and hypnosis (Ofshe and Singer, 1994), especially when protracted for a long period of time, may lead to false memory fabrication (Scoboria et al., 2017).

## “Give me your attention”: hypnosis and the brain

Hypnosis is an altered state of consciousness that can modulate both subjective experience (Rainville and Price, 2003) and physiological responses (Gruzelier, 1998). A high level of hypnotisability (that is: a high level of susceptibility to hypnosis; Rainville and Price, 2003; Vanhaudenhuyse et al., 2014) has been associated with the functional connectivity between the left dorsolateral prefrontal cortex and the dorsal anterior cingulate cortex (Faerman et al., 2024). Functional magnetic resonance (fMRI) under hypnosis has shown reduced connectivity between the executive control network, the default mode network, and the posterior cingulate cortex (Jiang et al., 2016) and a reduced activity of the dorsal anterior cingulate cortex (Jiang et al., 2016). Increased functional connectivity between the dorsolateral prefrontal cortex, the executive control network, and the insula in the salience network has also been observed (Jiang et al., 2016). Hypnotic states seem to induce a lower activation of the brainstem, of the right primary somatosensory cortex, and of the left and right insula when compared to wakefulness (Vanhaudenhuyse et al., 2009). Assessment of the hypnotic state during electroencephalography (EEG) has shown changes in brain oscillations, with increased theta band (indicating drowsiness) and changes in the gamma band (indicating problem solving, concentration; Jensen et al., 2015; Vanhaudenhuyse et al., 2014).

The use of hypnosis as therapy (known as hypnotherapy) seeks to induce a hypnotic state, which is then followed by suggestions aiming to positively modify a person's behavior (suggestion therapy; Karle and Boys, 1987) or to help them revive a repressed memory (regression therapy; Hunter, 2009) to correct maladaptive mental schemas (Alladin, 2013; Horowitz, 1988). To induce the hypnotic state, a hypnotic induction is generally used, which follows specific steps (Gruzelier, 1998) and may vary in length.

The applications of hypnosis in recalling memories cover different professional settings. Regression therapy (Hunter and Eimer, 2012) uses hypnosis to recall early life events which may have been purposely forgotten (repressed) as a defense mechanism to protect the self (Kramer, 2010). As these repressed memories may still operate outside of the person's conscious awareness, it is possible to experience maladaptive working schemas that may lead to a series of behavioral and mental problems (Mares, 2022). Helping the patient in recalling the forgotten event is expected to lead them to become aware of the memory, allowing for a rationalization of the event, acceptance, and eventual benefits to the person's mental health (Bateman et al., 2021). Hypnotic regression techniques have also been used to help eyewitnesses or crime victims in recalling memories of events; however, with several criticisms (Lynn et al., 2001; Winter, 2013). Additionally, hypnosis is used to enhance memory performance, usually employing post-hypnotic suggestions (suggestions made while the subject is in a hypnotic state, to be acted upon at some later time after the hypnosis session), such as hypnotic anchors (internal/external triggers—e.g., a gesture or a word—associated to a specific response; Schmidt et al., 2024b).

## A forgetful heart: the role of the cardiovascular system in memory and cognition

The central nervous system exerts control over the autonomic and neurohumoral regulation of the cardiovascular system (top-down regulation; Tahsili-Fahadan and Geocadin, 2017). Alterations of the brain-heart axis, such as the ones occurring in the heart-stroke syndrome, can induce autonomic dysfunctions that affect heart rate variability and baroreceptor reflex sensitivity (Scheitz et al., 2018). Moreover, psychological stress has been shown to influence the onset of several cardiovascular diseases (Dar et al., 2019; Esler, 2017; Leo et al., 2023). Vice versa, the influence that the heart exerts on the brain (bottom-up regulation) is also recognized (Taylor et al., 2010; Wolters et al., 2018).

Memory and cognition are largely impacted by reduced cerebral blood flow (Birdsill et al., 2013). Cerebral perfusion is a function of cardiac output, arterial stiffness, and cerebral autoregulation (Moore and Jefferson, 2021). A lower cardiac output is associated with smaller gray matter in older adults (Park et al., 2017) and with worse cognitive performance (Sabayan et al., 2015). Patients with heart failure (HF) exhibit a reduced volume of the hippocampus, which deeply affects cognitive functions (Frey et al., 2021; Lu et al., 2022). Moreover, HF is associated with a 60% increased risk of developing dementia (Wolters et al., 2018). Arterial stiffness can impact brain health, as a greater stiffness of the aorta increases the pulsatile energy to the periphery, detrimentally impacting high blood flow organs such as the brain (Moore and Jefferson, 2021). An increase in the carotid-femoral pulse wave velocity (PWV—a measurement of arterial stiffness) is associated with a reduction in the total brain volume in older adults (Sabayan et al., 2015). Hypertension is a major risk factor in the development of dementia, inducing disruption in cerebral autoregulation (Carnevale et al., 2012; Scullin et al., 2017; Walker et al., 2017) and cerebral blood flow (Jennings et al., 2017). A 20 mmHg increase in systolic blood pressure is associated with a 62% increased risk of developing vascular dementia in people aged 30 to 50 years of age (Emdin et al., 2017). Cognitive impairments and dementia increase susceptibility to the occurrence of false memories (Malone et al., 2019; Watson et al., 2001).

Cognitive-attentional functions are negatively influenced by increased sympathetic activation, which reduces cognitive flexibility due to body arousal (Critchley et al., 2013). An increase in the resting heart rate has been associated with an increased risk of cognitive decline (Kim et al., 2022). Heart rate variability (HRV), which is the variation in time interval between heartbeats usually measured by an electrocardiogram (ECG; Shaffer and Ginsberg, 2017), has been suggested as a physiological correlate of cognitive functioning (Forte et al., 2019), with higher HRV linked to better cognitive performance, and lower HRV linked to worse cognitive function (Forte et al., 2019). High HRV increases control over memory and helps suppress unwanted memories (Gillie et al., 2014), while low HRV worsens performance in short and long-term verbal memory (Frewen et al., 2013). Possible explanations can be found in the relationship between resting HRV and active-inhibitory prefrontal-subcortical circuits, with a higher resting HRV related to increased activity of the executive brain regions (Forte et al., 2019; Thayer et al., 2012) and a lower resting HRV related to a hypoactive prefrontal regulation (Forte et al., 2019; Park and Thayer, 2014). A higher resting-state HRV has been associated with greater memory retrieval functions (Williams et al., 2019). On the contrary, subjects with a lower resting-state HRV have shown to be less capable of discriminating true from false memories (Feeling et al., 2021).

Cerebral hemorrhage can also affect episodic memory, leading to the occurrence of false memories formation (confabulations). Damages to the hippocampus or the temporal lobe can cause retrograde amnesia (Ketonis et al., 2024). Ischemic stroke is a strong risk factor in the development of dementia (Kuzma et al., 2018), due to lesions to cortical and subcortical areas that mediate executive functions (Kalaria et al., 2016). Confabulations have been commonly reported subsequent to traumatic brain injuries (Demery et al., 2001; Dockree et al., 2006) or other cerebrovascular incidents (Dalla Barba et al., 1997; DeLuca and Cicerone, 1991; Parand et al., 2020).

## “Follow the beat”: how hypnosis affects the cardiovascular system

**Figure 1:**
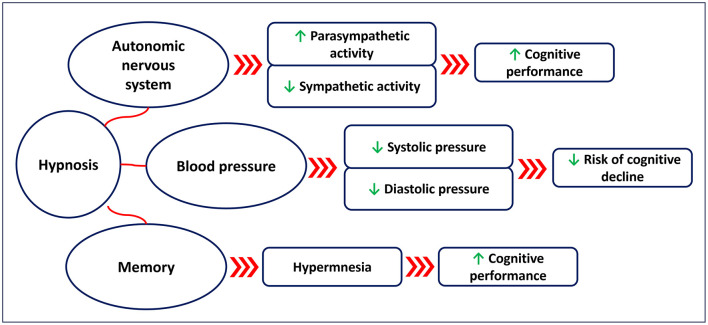
Suggested effects of hypnosis on the autonomic nervous system (changes in heart rate variability), blood pressure, and memory.

Hypnosis has been shown to affect the cardiovascular system in terms of HR and blood pressure (Figure 1). Several studies (Aubert et al., 2009; Bello et al., 2019; Boselli et al., 2018; De Benedittis, 2024; DeBenedittis et al., 1994; Diamond et al., 2007; Hippel et al., 2001; Kekecs et al., 2016; VandeVusse et al., 2010) have assessed the autonomic nervous system response during hypnosis, showing an increase in parasympathetic activity and a reduction in sympathetic activity. A randomized controlled study (Kekecs et al., 2016) conducted on 121 young adults showed that hypnosis was effective in reducing tonic sympathetic nervous activity (measured with skin conductance level) compared to the non-hypnosis control. Another study (DeBenedittis et al., 1994) conducted on 10 healthy subjects showed that hypnosis affects HRV, with increased parasympathetic activation and reduced sympathetic activity. A quasi experimental-study (VandeVusse et al., 2010) conducted on a sample of 30 healthy women showed that parasympathetic activity (changes in HRV) was enhanced during hypnosis. A study (Aubert et al., 2009) conducted on 12 healthy subjects undergoing ECG at rest and during hypnosis showed an enhanced parasympathetic activation (changes in HRV) during the hypnotic state. A quasi-experimental study (Bello et al., 2019) conducted on 15 healthy young men showed that hypnosis induces an increase in HRV. A study (Diamond et al., 2007) conducted on 10 healthy subjects showed that the high frequency of HRV is positively correlated with the depth of the hypnotic state. Another study (Hippel et al., 2001) conducted on 10 healthy subjects showed that hypnosis is effective in reducing sympathetic activity (changes in HRV). A prospective observational study (Boselli et al., 2018) conducted on 40 healthy subjects showed that hypnosis was effective in increasing the parasympathetic tone (assessed using the Analgesia/Nociception Index).

Hypnosis has also been shown to affect blood pressure. A study (Emdin et al., 1996) conducted on 10 highly hypnotisable subjects undergoing ECG and blood pressure monitoring during a 50 min hypnosis session showed that hypnosis induced bradycardia (*P* = 0.04) and a light increase in systolic (*P* = 0.01) and diastolic (*P* = 0.03) blood pressure. A randomized controlled pilot study (Raskin et al., 1999) conducted on 33 patients with hypertension, showed that self-hypnosis practiced twice a day for 1 month was effective in reducing diastolic blood pressure (*P* < 0.05) in the intervention group compared to the non-hypnosis control. A randomized controlled trial (Gay, 2007) conducted on 30 participants with mild essential hypertension, showed that 8 × 30 min sessions of hypnosis were effective in reducing diastolic blood pressure in the intervention group post-treatment (*P* < 0.0001) as well as at 6 (*P* < 0.003) and 12-month (*P* < 0.003) follow-up, compared to the non-hypnosis control. Reduction in systolic blood pressure was also observed in the intervention group post-treatment (*P* < 0.0003), and at 6 (*P* < 0.001) and 12-month (*P* < 0.001) follow-up, compared to the non-hypnosis control. A non-randomized control study (Holdevici and Crǎciun, 2013) conducted on 80 participants diagnosed with primary and secondary hypertension who completed an 8-month Ericksonian hypnosis treatment showed that hypnosis was effective in decreasing stress [Perceived Stress Scale (Cohen et al., 1983)—*P* = 0.003] and in improving quality of life [SF-36 (Ware and Sherbourne, 1992) —*P* < 0.05] of these patients post-intervention. However, the number of studies that have investigated the effects of hypnosis on hypertension is still low, and further research is needed to better clarify at what degree hypnosis is able to affect blood pressure, and the physiological mechanisms behind it.

## “Now I remember!”: memory and hypnosis

Hypnosis has been suggested as a potential tool to enhance memory and learning. This may be partially explained by the effects that hypnosis has in reducing sympathetic activity (Aubert et al., 2009; Fernandez et al., 2021; Kekecs et al., 2016; Yüksel et al., 2013) and thus in favoring parasympathetic activity, with the latter related to better cognitive performance (Critchley et al., 2013). A randomized controlled study (Çetin et al., 2016) conducted on 70 healthy participants showed that hypnosis was more effective in vocabulary learning for a second language compared to the non-hypnotized control group. Another study (Nemeth et al., 2013) conducted on 14 university students showed that hypnosis has a positive effect on learning. A randomized controlled study (Lindeløv et al., 2017) conducted on 52 participants with brain injury showed the positive effects of a four-week hypnosis intervention (1 h/week) in improving memory performance in this population. Another randomized controlled study (Fligstein et al., 1998) conducted on 60 university students who were asked to recall the content of 60 slides at three recall periods showed that the hypnosis group recalled more correct items than the non-hypnosis group. A recent study (Schmidt et al., 2024a) conducted on 24 participants showed that a post-hypnotic anchor was effective in improving memory recall, with the effect also lasting at 1-week follow-up. A pilot study (Duff and Nightingale, 2005) conducted on seven patients with dementia reported a positive effect of hypnosis on memory after a 9-month intervention, with benefits also maintained at 12-month follow-up (Duff and Nightingale, 2006). Several studies have demonstrated the hypnotic hypermnesia effect in laboratory studies where individuals were administered hypnosis to help recall previous visually presented material (Crawford and Allen, 1983; Kunzendorf et al., 1987; McConkey and Kinoshita, 1988; Stager and Lundy, 1985). This seems to be especially true in highly hypnotisable individuals (Crawford and Allen, 1983). However, the findings of one (Stager and Lundy, 1985) of these studies reporting enhanced memory after hypnosis could not be replicated by another study (Lytle and Lundy, 1988). Moreover, several other studies have shown no effects of hypnosis in enhancing memory (Baker et al., 1983; Dasgupta et al., 1994; Dinges et al., 1992; Dywan, 1988; Nogrady et al., 1985; Putnam, 1979; Register and Kihlstrom, 1987). It has been argued that the hypermnesia noted in some studies involving hypnotic techniques is not induced by hypnosis per se but rather induced by the repeated retrieval effort (Erdelyi, 1994).

Conversely, hypnosis has also been suggested to induce functional amnesia, similar to the one observed in dissociative episodes (Kihlstrom, 1979, 1997). Post-hypnotic amnesia refers to the difficulty of a person in remembering the experience they had during hypnosis (Kihlstrom and Evans, 2014); however, this effect is reversible when a prearranged cue is present (Kihlstrom and Evans, 2014). Post-hypnotic amnesia seems to be associated with hypnotic-induced interferences in the temporal sequencing during the memory recall process (Kihlstrom and Evans, 2014), which may be partially explained by the decoupling of the dorsolateral prefrontal cortex from the default mode networks (Jiang et al., 2016). Two types of post-hypnotic amnesia have been suggested (Evans and Thorn, 1966): (i) post-hypnotic recall amnesia, where the individual is unable to recall the events that occurred during hypnosis, and (ii) post-hypnotic source amnesia, where the individual remembers the information presented during hypnosis, but is unable to recall how they learned it. Post-hypnotic amnesia seems to mainly affect explicit (declarative) memory rather than implicit (non-declarative) memory (David et al., 2000; Kihlstrom, 1997, 2021). In very high hypnotisable individuals, post-hypnotic amnesia can be induced for material learned either before or during hypnosis (Barnier et al., 2001; Bryant et al., 1999). In some individuals, post-hypnotic amnesia cannot be broken down (before its reversal) even when they are exposed to a videotape playback of the events that occurred during hypnosis (McConkey and Sheehan, 1981; McConkey et al., 1980).

Hypnotic age regression has been suggested as a tool to recover early childhood memories. However, the most obvious limitation of this approach is that it is difficult to evaluate whether the memory recalled by the subject is an accurate description of a real-life event or a fabrication (Lynn and Kirsch, 1996; Spanos et al., 1994). This is especially true when considering that memory recall itself is not an accurate process (Schacter et al., 2011), as already discussed. Research on hypnotic regression is scarce and mainly based on anecdotal evidence. A study (Fromm, 1970) published in 1969 presented the case of a patient who was successful in recovering repressed childhood language using hypnotic regression. Context and expectations toward hypnosis have been shown to influence the response in recalling autobiographical memories, also enhancing the subjects' confidence in the accuracy of the memory, especially in highly hypnotisable subjects (Green, 1999). Hypnotic regression has been, and still is, a source of controversy, as its applications have been deemed to potentially induce false memories (Ofshe and Singer, 1994), strongly affecting the outcome of the treatment (from both a therapeutic and a legal perspective—such as in alleged cases of abuse). It has been suggested that imagery and unintentional suggestions may lead to the formation of false memories (Arbuthnott et al., 2001; Strange and Takarangi, 2015) due to the source misattribution effect (Schacter, 1999). Additionally, hypnotic suggestions can inflate the person's confidence of the accuracy of their own (false) memories (imagery inflation), strongly affecting the rewriting of their memory (Heaps and Nash, 1999; Schacter, 1999; Wagstaff et al., 2004; Whitehouse et al., 1988). Some studies have argued that it is a high hypnotisability level rather than hypnosis per se that induces false memories (Barnier and McConkey, 1992; Bryant and Barnier, 1999; Sheehan et al., 1991). But opposing views have been presented as well (Wagstaff et al., 2011). Additionally, a study (Ready et al., 1997) on hypnotic memory recall has shown the negative impact of emotional distress (i.e., anxiety) in producing inaccurate memories, with highly anxious subjects more inclined to inaccurate recalling than non-anxious ones. Hypnotic suggestions have shown the potential to intentionally (Gravitz, 1994; Sheehan et al., 1984; Terrance et al., 2000) and unintentionally (Robin et al., 2018) modify memory recall. Pre-hypnotic warnings and post-hypnotic suggestions may partially reduce the occurrence of false memories (Wagstaff et al., 2008); however, this has been debated (Green et al., 1998).

## Connecting the dots: the role of hypnosis on cardiovascular and cognitive functions and its implications for memory enhancement

The occurrence of false memories is related to a series of cognitive and physiological factors, each playing its part in fabricating a memory of an event that never occurred. Errors in the memory processes and emotional distress have an important role in false memory formation (Hertel and Brozovich, 2010; Straube, 2012). Social pressure, personal expectancy, misleading information, leading questions, and imagery inflation can all contribute to false memory formation (Garry et al., 1996; Hirt et al., 1999; Loftus, 1975; Reysen, 2007). In addition, psychoactive drugs and sleep deprivation can contribute to errors in the memory processes and to the occurrence of false memories (Kloft et al., 2023). Furthermore, cardiovascular factors associated with the interruption or reduction in blood flow to the brain (Birdsill et al., 2013) may lead to subsequent structural brain changes (Park et al., 2017), contributing to memory impairments and confabulation (DeLuca and Cicerone, 1991).

While hypnosis has been deemed as one of the causes of false memory fabrication, it is also true that it has shown relevant beneficial effects on both cognitive and cardiovascular functions. Hypnosis seems to positively affect the cardiovascular system with both direct and indirect effects. Direct effects relate to the influence of hypnosis on the autonomic nervous system (Aubert et al., 2009; Bello et al., 2019; Boselli et al., 2018; De Benedittis, 2024; DeBenedittis et al., 1994; Diamond et al., 2007; Hippel et al., 2001; Kekecs et al., 2016; VandeVusse et al., 2010), which regulates heart rate and blood pressure. Relaxation techniques have been shown to be effective in increasing parasympathetic activity while reducing sympathetic activity (Terathongkum and Pickler, 2004). During a hypnotic session, relaxation is usually accomplished during the initial phases of the hypnotic induction, where techniques such as progressive muscle relaxation and emphasis on focused breathing are employed (Karle and Boys, 2010). The decrease in sympathetic activity induced by hypnosis also mitigates the response of the cardiovascular system to emotional distress (Leo et al., 2024), with suggested positive effects on the heart and in reducing the incidence of conditions related to increased sympathetic activity and decreased parasympathetic activity (e.g., arrhythmias, hypertension). The indirect effects of hypnosis on the cardiovascular system relate to its contribution to inducing behavioral changes effectively, leading to the uptake of a healthier lifestyle (e.g., quitting smoking, reduced snacking, increased exercise adherence; Carmody et al., 2008; Delestre et al., 2022; Milling et al., 2018), therefore reducing the risk factors associated with cardiovascular diseases. Behavioral changes are often reached using hypnotic and post-hypnotic suggestions (e.g., ego-strengthening, anchoring; Karle and Boys, 2010). As brain health and cognitive performance depend on cardiovascular functions (such as adequate blood flow to the brain; Launer et al., 2015; Moroni et al., 2018), it seems clear that improved cardiovascular health can positively affect the brain, reducing the risk of early cognitive decline and memory deficit.

The role of hypnosis in improving cognitive functions has also been discussed, with contrasting results. The positive impact of hypnosis on cognitive functions may be related to several factors, such as increased relaxation and reduced sympathetic activity. Increased relaxation can improve focused attention and the recalling of episodic memory (Xu et al., 2014). Practices such as meditation and mindfulness, which share with hypnosis the focus on a relaxed state, have been shown to be effective in memory recalling and memory enhancement (Basso et al., 2019; Heeren et al., 2009; Subramanya and Telles, 2009), possibly suggesting that it is the relaxed state itself and not the technique per se that benefits memory. A decrease in sympathetic activity during the hypnotic state helps in reducing emotional distress that negatively affects memory functions (Shields et al., 2017). The use of imagery is a fundamental part of the hypnotic process, with imagery of emotional events capable of activating the autonomic nervous system in a similar way in which it is activated while experiencing the event in real life (Kosslyn et al., 2001). Guided imagery, when used as a tool to increase relaxation, may help improve memory recall. Self-imagining has been suggested as a potential tool to enhance memory in memory-impaired individuals (Grilli and Glisky, 2010; Raffard et al., 2016). However, several concerns about the use of hypnosis to enhance memory functions have also been raised. The hypermnesia induced by hypnosis reported by some studies (Crawford and Allen, 1983; Kunzendorf et al., 1987; McConkey and Kinoshita, 1988; Stager and Lundy, 1985) has been debated by other authors (Baker et al., 1983; Dasgupta et al., 1994; Dinges et al., 1992; Dywan, 1988; Nogrady et al., 1985; Putnam, 1979; Register and Kihlstrom, 1987), arguing that the observed enhancement of memory performance was more likely related to the repeated retrieval effort made by the participants (Erdelyi, 1994). Moreover, despite the fact that guided imagery is often used with the aim of reconstructing the memory of an event/situation (Arbuthnott et al., 2001), it can also negatively affect memory recall by facilitating the formation of false memories (Arbuthnott et al., 2001; Kealy and Arbuthnott, 2003; Paddock and Terranova, 2001), with realistic imagery more inclined to produce false memories compared to metaphoric imagery (Arbuthnott et al., 2001). Memory distortion during guided imagery is not exclusive to hypnosis but is common to several psychotherapeutic contexts where imagination is encouraged (Loftus, 1996; Lynn and Kirsch, 1996). Therefore, particular attention should be given to avoid leading the patient during the process when using guided imagery for memory recall during hypnosis or other psychotherapeutic approaches.

In summary, while the physiological effects of hypnosis may have a positive role on memory functions due to their beneficial impact on the cardiovascular system, the use of some hypnotic techniques, such as guided imagery, can increase the risk of developing false memories and should be employed with caution.

## Conclusion and further directions

Human memory is not an exact record of past experiences but an adaptive process inclined to errors. Several factors may interfere with memory recall, potentially leading to incorrect memories. Factors such as emotional and physical stress can alter memory performance and recall. The brain-heart interaction is crucial in preserving brain health and improving cognitive processes. Health conditions affecting the heart can disrupt the balance between heart and brain processes, leading to cognitive decline and memory impairments. Additionally, alterations in the cardiovascular system may increase the occurrence of incorrect memory recall. Hypnosis has been shown to affect heart rate variability and blood pressure, suggesting a potential role in preventing cardiovascular diseases related to increased sympathetic activity and decreased parasympathetic activity. However, the paucity of evidence on the role that hypnosis has on the cardiovascular system leaves several unanswered questions. Contradictory findings on the role that hypnosis has on memory and cognitive processes make it difficult to draw proper conclusions. Moreover, guided imagery techniques used to enhance the recall of autobiographical events may lead to memory distortions (incorrect recall or source misattribution), and particular attention should be given to avoid unintentional hypnotic suggestions that could induce false memories. Cautions should be exerted when conducting regression therapy, and such an approach should be evaluated case by case.

The large heterogeneity in study design and hypnotic interventions of the studies reviewed in our paper may have been a critical factor for the differences in results reported in literature. Additionally, not all the examined studies have screened for hypnotisability level, a factor that may have contributed to the contradictory results.

Further research should be carried out to better define the effects of hypnosis on the brain and the cardiovascular system, as well as its impact on cognitive processes. Studies with robust design (e.g., randomized controlled studies) and bigger sample size should be conducted to test the efficacy of hypnosis in memory enhancement and investigate its potential beneficial effects in preventing cardiovascular diseases that increase the risk of cognitive decline (e.g., hypertension).
